# Supplementary material for: Structural plasticity of KIR2DL2 and KIR2DL3 enables altered docking geometries atop HLA-C
Source: Nat Commun. 2021 Apr 12;12:2173. doi: 10.1038/s41467-021-22359-x (PMC8041999; doi:10.1038/s41467-021-22359-x)
Supplement: Supplementary file 1 — Supplementary Information [file 41467_2021_22359_MOESM1_ESM.pdf]

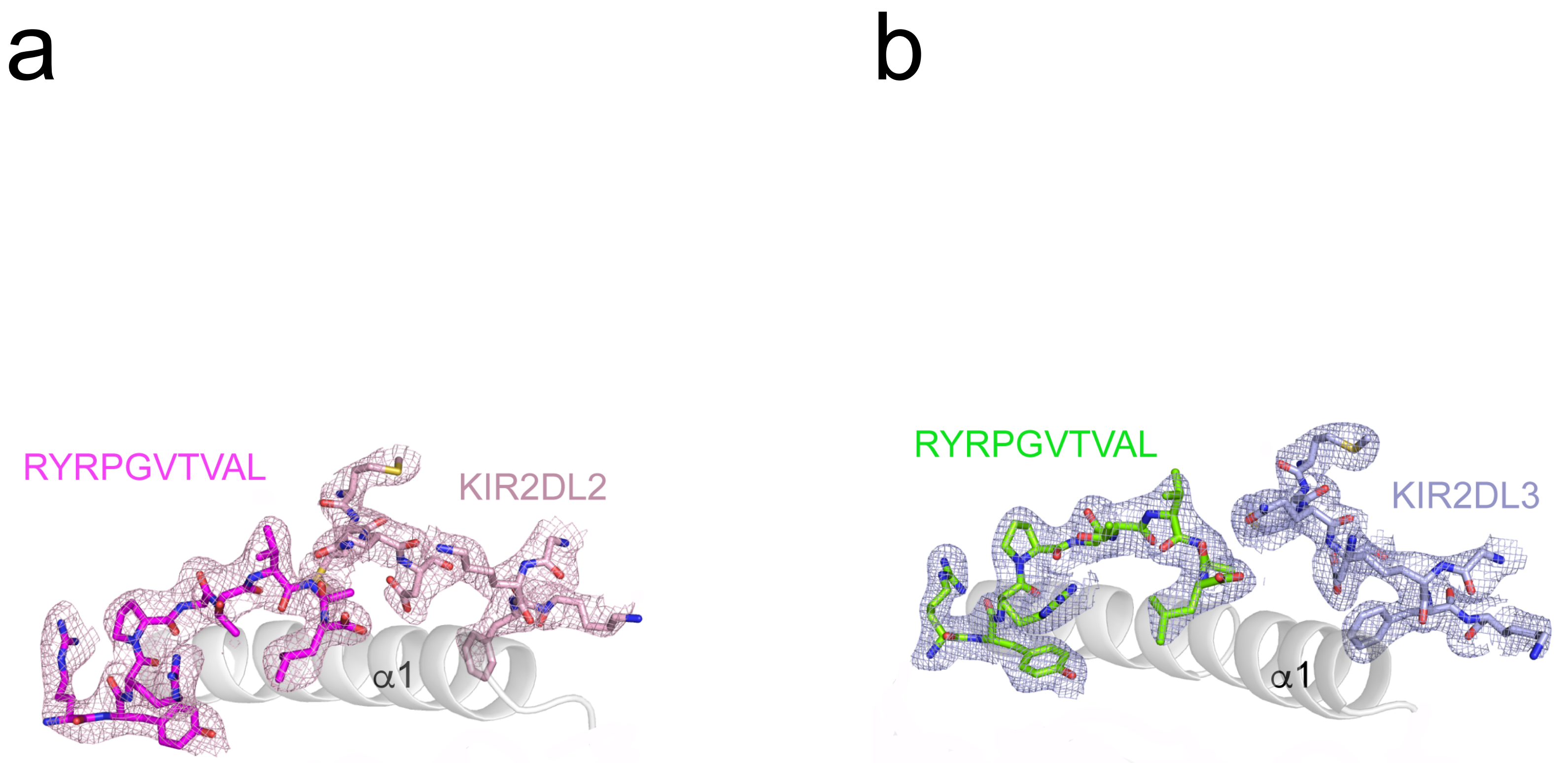

Supplementary Figure 1. Representative electron density for the KIR2DL2-HLA-C*07:02-RL9 and KIR2DL3-HLA-C*07:02-RL9 complexes. The electron density (shown as mesh) for the RL9 peptide and the C-C' loop of KIR2DL2 (a) and KIR2DL3 (b) are shown from their resepective complexes with HLA-C*07:02-RL9.

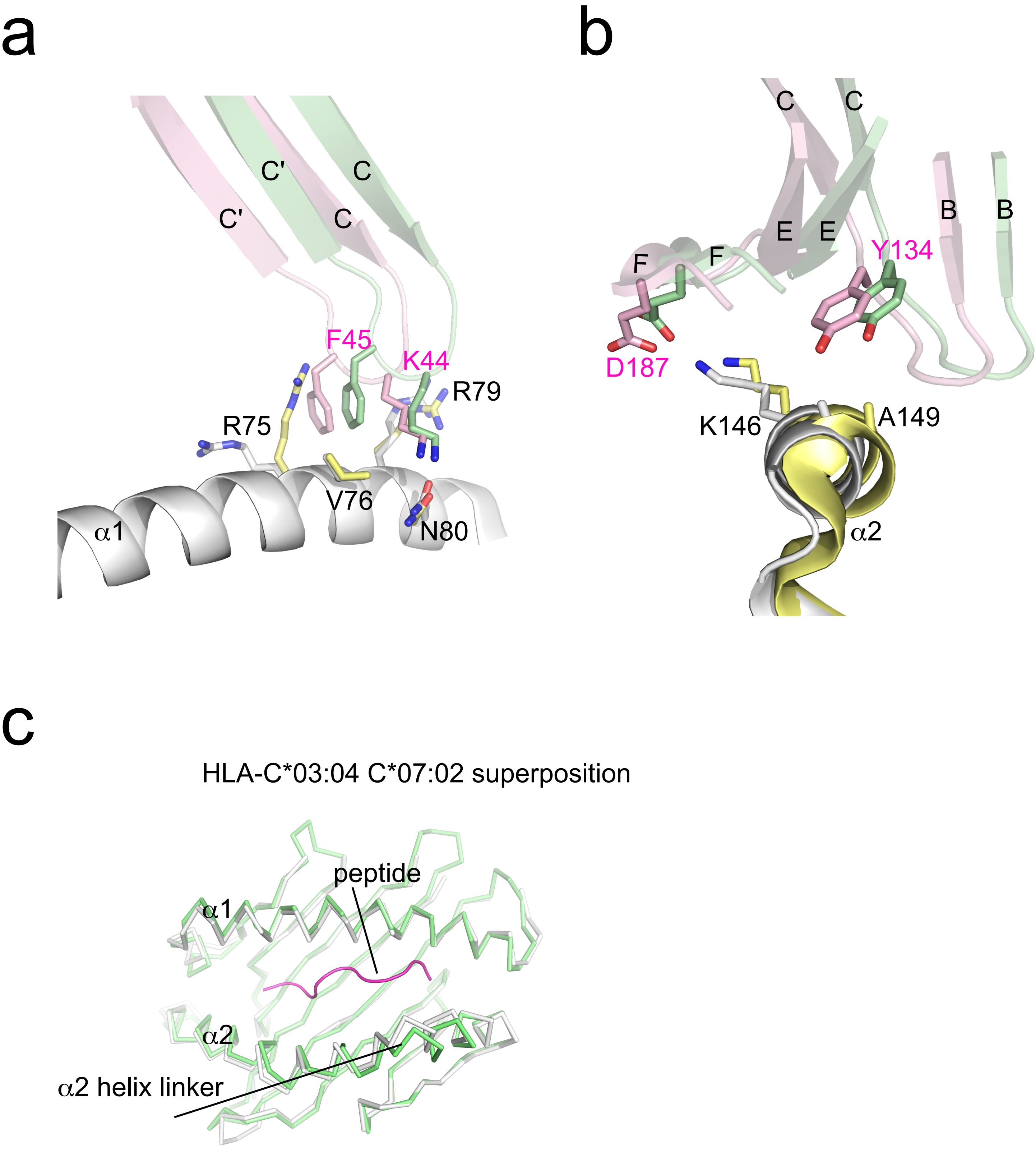

Supplementary Figure 2. Differences in KIR2DL2 mediated contacts to HLA-C*03:04-GL9 and C*07:02-RL9. KIR2DL2 ternary complexes represented as cartoon, with the HLA-C*03:04-GL9 complex coloured green and the C*07:02-RL9 complex coloured pink. (a) A representation of the contacts formed to HLA-C*07:02-RL9 that are not observed in C*03:04-GL9. (b) A representation of the contacts formed to HLA-C*03:04-GL9 that are not observed in C*07:02-RL9. (c) Superposition of the structures of HLA-C*07:02-RL9 (grey) and HLAC*03:04-GL9 (green) in complex with KIR2DL2. Depicted as Cα trace. The wider spacing of HLAC*03:04 at the α2 helical hinge is higlighted.

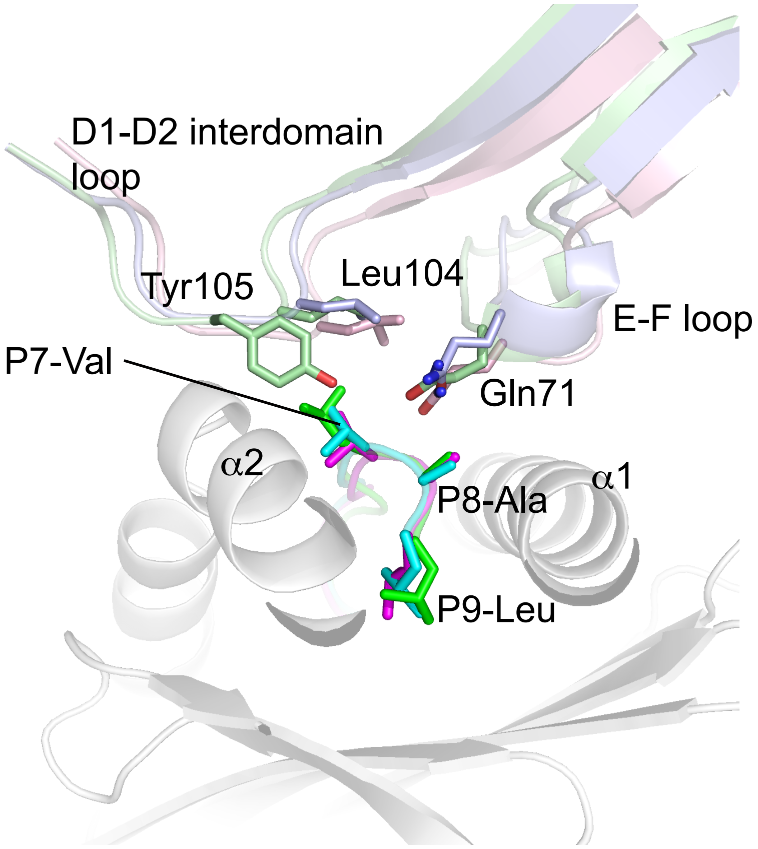

Supplementary Figure 3. Contacts between KIR2DL2/2DL3 and the peptides presented by HLA-C*03:04-GL9 and C*07:02-RL9. The ternary complexes of KIR2DL2/2DL3 bound to HLA-C*03:04-GL9 and C*07:02-RL9 are represented as cartoon. KIR2DL2-C*03:04-GL9 is coloured green, KIR2DL2-C*07:02-RL9 is coloured pink, KIR2DL3-C*07:02-RL9 is coloured blue.

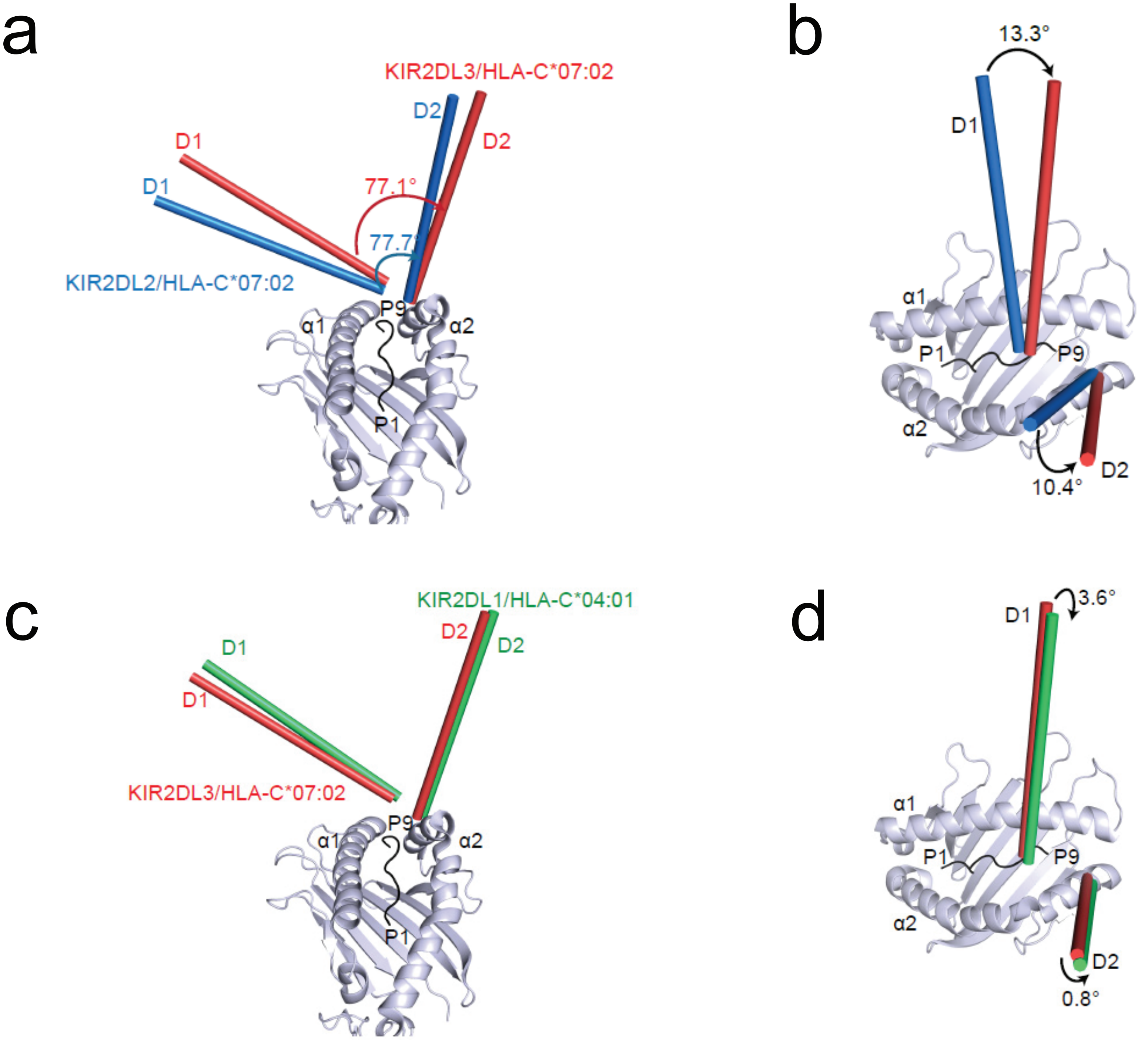

Supplementary Figure 4. Comparison of the docking angles of KIR2DL1, 2DL2 and 2DL3 on HLA-C. The D1 and D2 domains of the KIR are represented as principal axes, the HLA and peptides are represented in cartoon format. (a and b) Comparison of KIR2DL2 (blue) and 2DL3 (red) docking angles on HLA-C*07:02-RL9. (c and d) Comparison of KIR2DL3 (red) docking angles on HLA-C*07:02-RL9 against 2DL1 (green) on HLA-C*04:01.

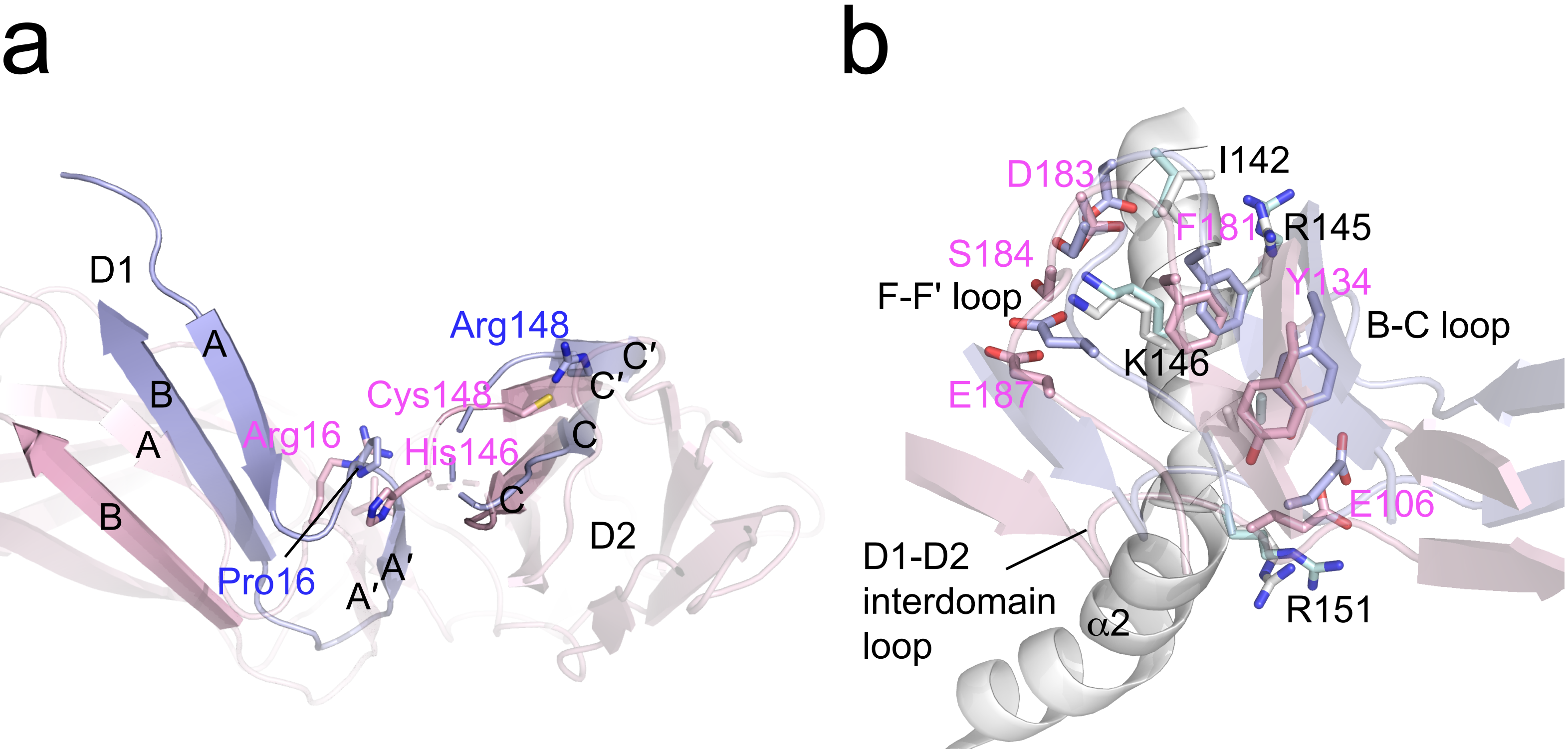

Supplementary Figure 5. Differences between KIR2DL2 and KIR2DL3 docking to the α2-domain of HLA-C*07:02-RL9. Comparison of KIR2DL2 (pink) and KIR2DL3 (blue) D2 contacts to the α2 helix of HLA-C*07:02-RL9 (KIR2DL2 contacts coloured grey, KIR2DL3 contacts coloured cyan). The D1-D2 linker loop, the B-C loop and F-F′ loops are highlighted.

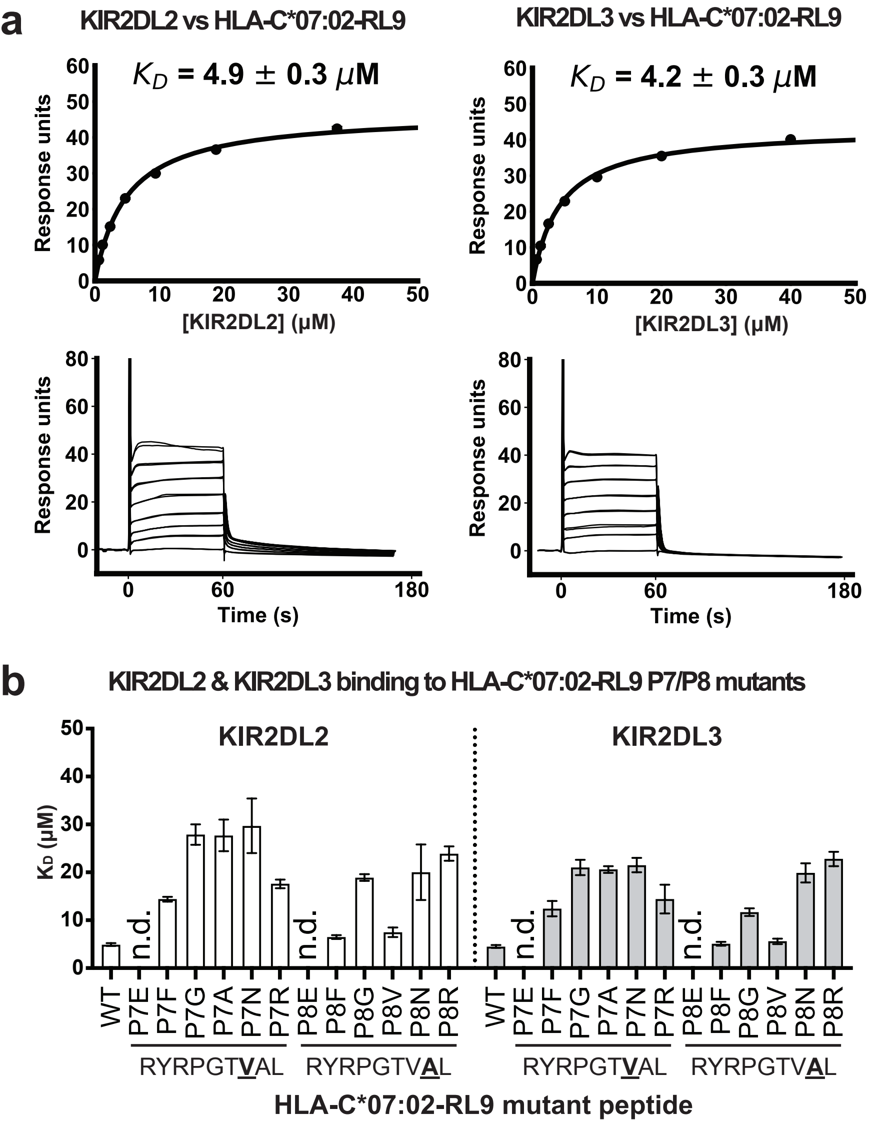
a
KIR2DL2 vs HLA-C*07:02-RL9
KIR2DL3 vs HLA-C*07:02-RL9
K_D = 4.9 ± 0.3 μM
K_D = 4.2 ± 0.3 μM
Response units
[KIR2DL2] (μM)
[KIR2DL3] (μM)
Time (s)
b
KIR2DL2 & KIR2DL3 binding to HLA-C*07:02-RL9 P7/P8 mutants
KIR2DL2
KIR2DL3
K_D (μM)
n.d.
WT
P7E
P7F
P7G
P7A
P7N
P7R
P8E
P8F
P8G
P8V
P8N
P8R
RYRPGTVAL
HLA-C*07:02-RL9 mutant peptide

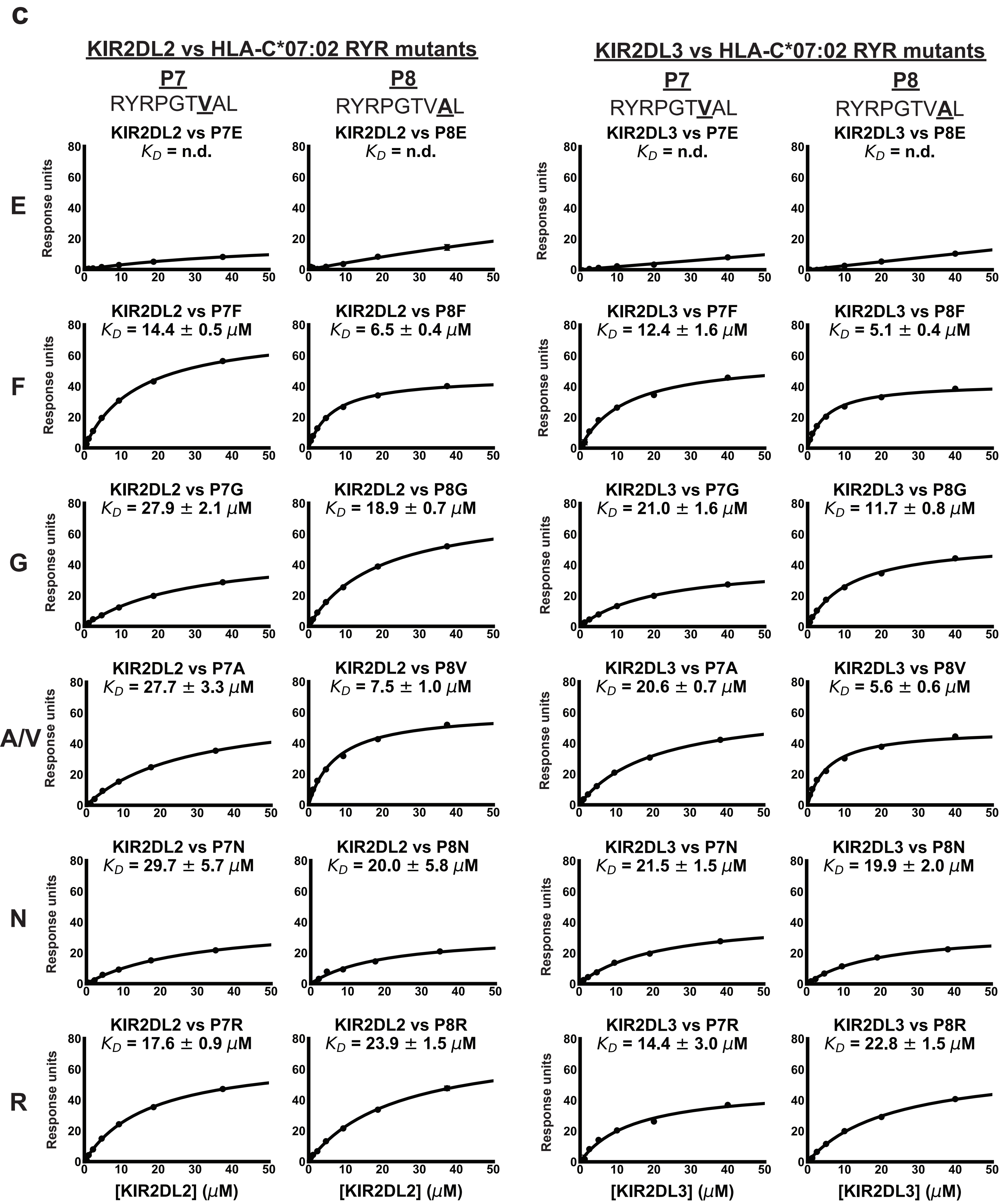
c
KIR2DL2 vs HLA-C*07:02 RYR mutants
KIR2DL3 vs HLA-C*07:02 RYR mutants
P7
RYRPGTVAL
P8
RYRPGTVAL
E
F
G
A/V
N
R
KIR2DL2 vs P7E
K_D = n.d.
KIR2DL2 vs P8E
K_D = n.d.
KIR2DL3 vs P7E
K_D = n.d.
KIR2DL3 vs P8E
K_D = n.d.
KIR2DL2 vs P7F
K_D = 14.4 ± 0.5 μM
KIR2DL2 vs P8F
K_D = 6.5 ± 0.4 μM
KIR2DL3 vs P7F
K_D = 12.4 ± 1.6 μM
KIR2DL3 vs P8F
K_D = 5.1 ± 0.4 μM
KIR2DL2 vs P7G
K_D = 27.9 ± 2.1 μM
KIR2DL2 vs P8G
K_D = 18.9 ± 0.7 μM
KIR2DL3 vs P7G
K_D = 21.0 ± 1.6 μM
KIR2DL3 vs P8G
K_D = 11.7 ± 0.8 μM
KIR2DL2 vs P7A
K_D = 27.7 ± 3.3 μM
KIR2DL2 vs P8V
K_D = 7.5 ± 1.0 μM
KIR2DL3 vs P7A
K_D = 20.6 ± 0.7 μM
KIR2DL3 vs P8V
K_D = 5.6 ± 0.6 μM
KIR2DL2 vs P7N
K_D = 29.7 ± 5.7 μM
KIR2DL2 vs P8N
K_D = 20.0 ± 5.8 μM
KIR2DL3 vs P7N
K_D = 21.5 ± 1.5 μM
KIR2DL3 vs P8N
K_D = 19.9 ± 2.0 μM
KIR2DL2 vs P7R
K_D = 17.6 ± 0.9 μM
KIR2DL2 vs P8R
K_D = 23.9 ± 1.5 μM
KIR2DL3 vs P7R
K_D = 14.4 ± 3.0 μM
KIR2DL3 vs P8R
K_D = 22.8 ± 1.5 μM
Response units
[KIR2DL2] (μM)
[KIR2DL3] (μM)

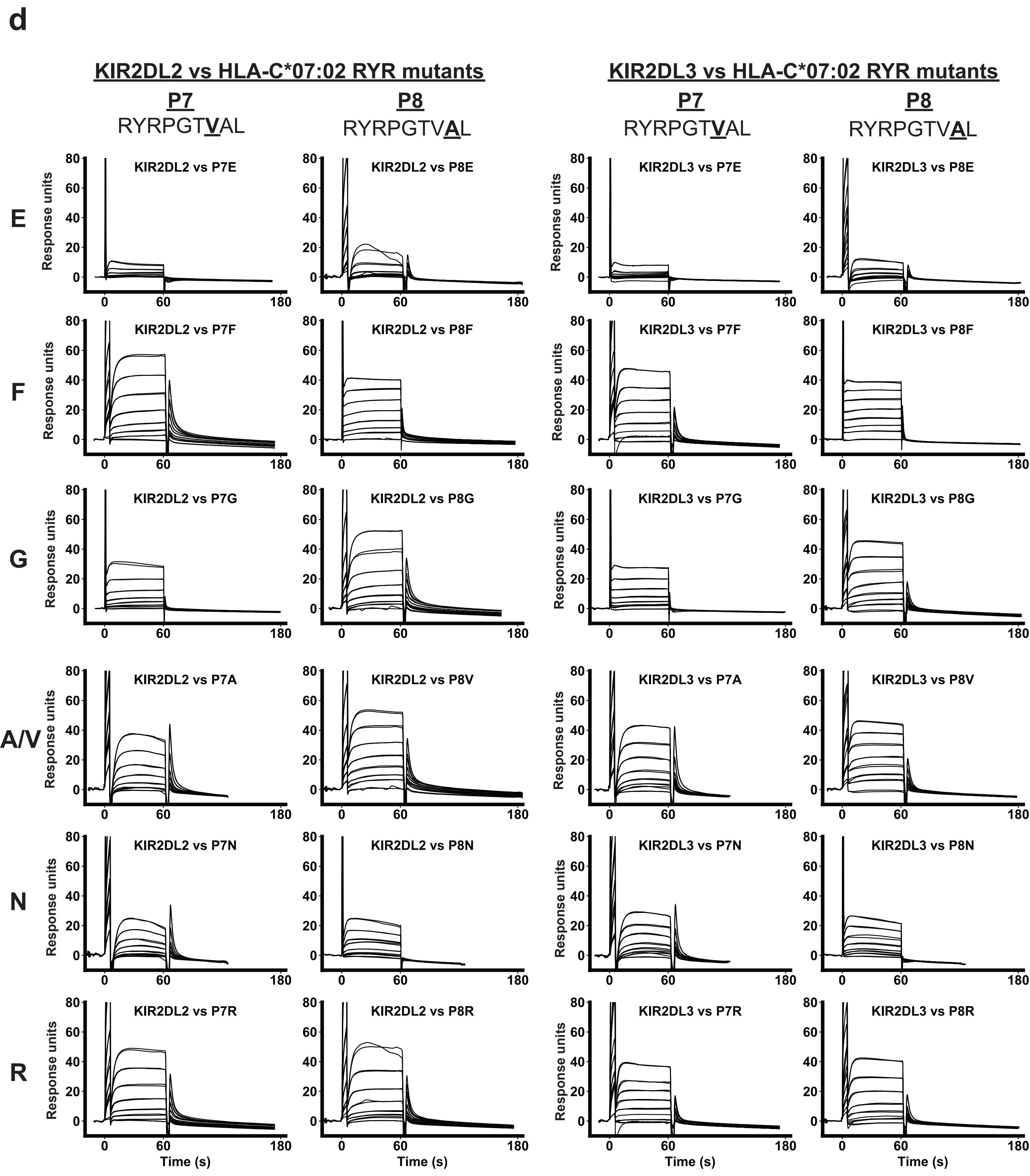

Supplementary Figure 6. Surface plasmon resonance measurements of KIR2DL2 and KIR2DL3 binding to HLA-C*07:02-RL9 mutant peptides. (a) Equilibrium binding analysis (top) of KIR2DL binding to RL9-WT. Mean response at each analyte concentration (circles), standard deviation between n=2 injections (error bars) and non-linear regression curve fit of the one-to-one specific binding model (line) are shown. Calculated KD value ± standard deviation is denoted inset. Corresponding reference-subtracted sensograms are also shown (bottom). Calculated KD values derived from n=2 injections and representative of n=2 independent experiments. (b) Summary of comparative binding of KIR2DL2 and KIR2DL3 to all HLA-C*07:02-RL9 P7 and P8 mutant peptides tested. Calculated KD value (bar lines) ± standard deviation of calculated KD values (error bars) are plotted. Calculated KD values derived from n=2 injections and representative of n=2 independent experiments. (c) Equilibrium binding analysis for data described in (b). (d) Corresponding reference-subtracted sensograms for data described in (b and c).

.

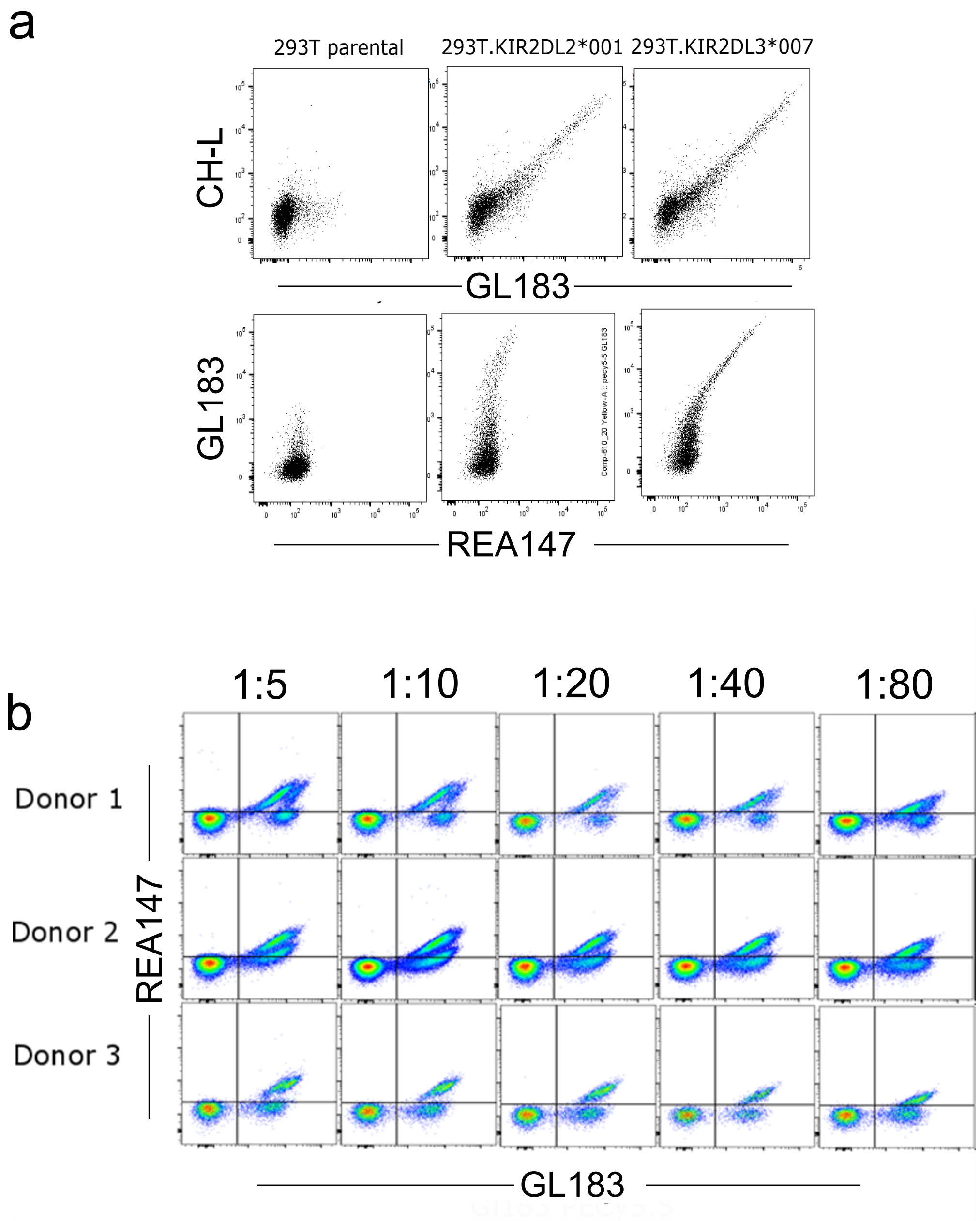

Supplementary Figure 7. Assessment of antibody staining. (a) 293T cells were transfected with plasmids encoding KIR2DL2*001 or KIR2DL3*007 and were stained with corresponding antibodies, GL183, REA147 and CH-L. (b) Different staining dilutions of REA147 antibody used to stain NK cells from three donors.

.

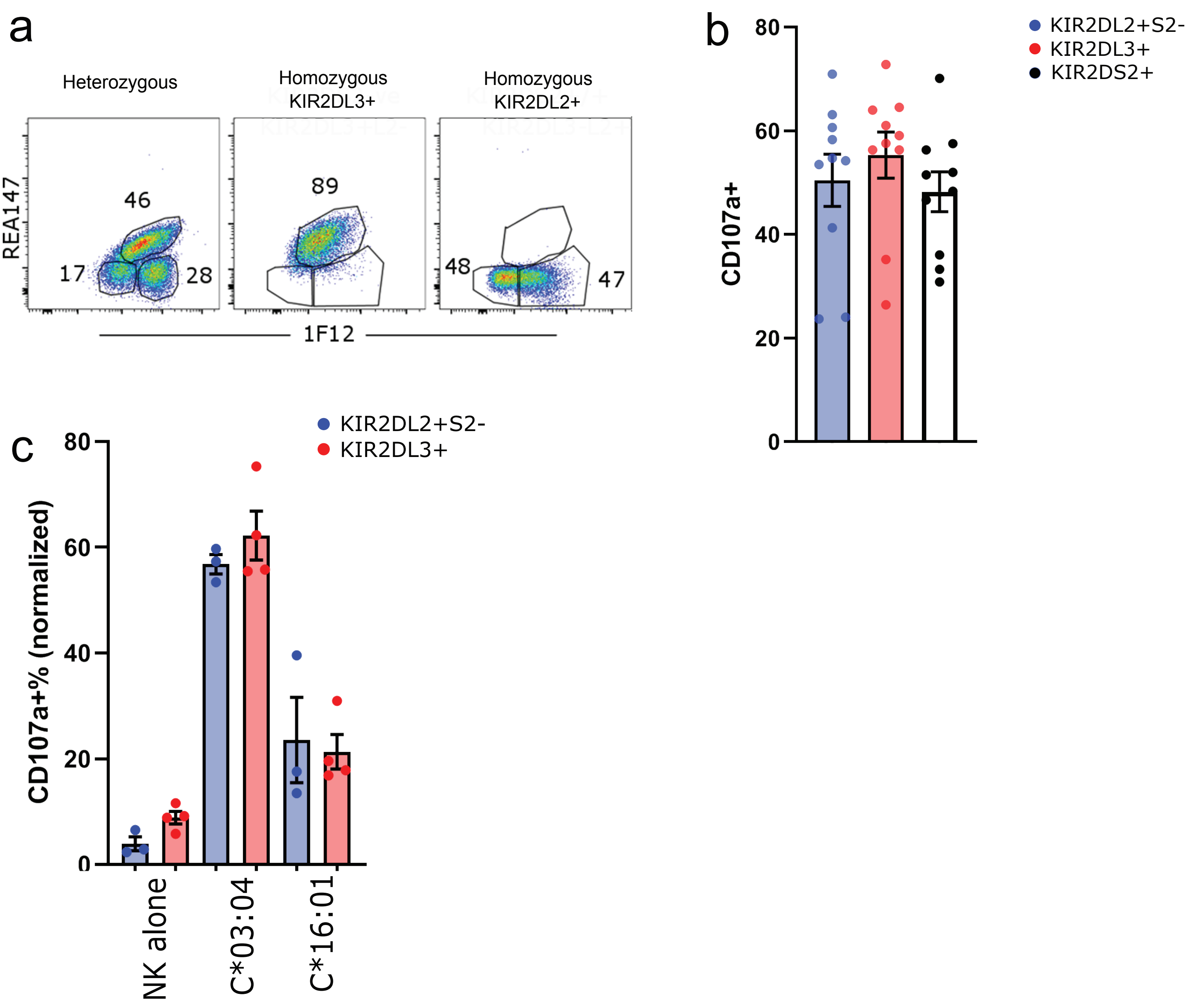

Supplementary Figure 8. Identification and functional analyses of KIR2DL2+/KIR2DS2- NK cells. (a) Flow cytometry plots of NK cells from donors heterozygous and homozygous for KIR2DL2/S2 or KIR2DL3, stained with GL183, REA147 and 1F12. Data shown is gated on GL183+ NK cells and excluding KIR2DL1/S1+ cells. (b) The proportion of CD107a+ cells within three subsets of NK cells in response to 721.221 cells; Data is from 10 heterozygous donors (n=10). (c) (c) Degranulation responses of homozygous donors were assessed following co-culture with either un-trans- fected 221 cells or 221 cells transfected with HLA-C*03:04 or -C*16:01 (n=3 for KIR2DL2+S2- and n=4 for KIR2DL3+ populations). Data shown is normalised to the response to untransfected 221 cells. Errors bars are mean values +/- SEM.

.

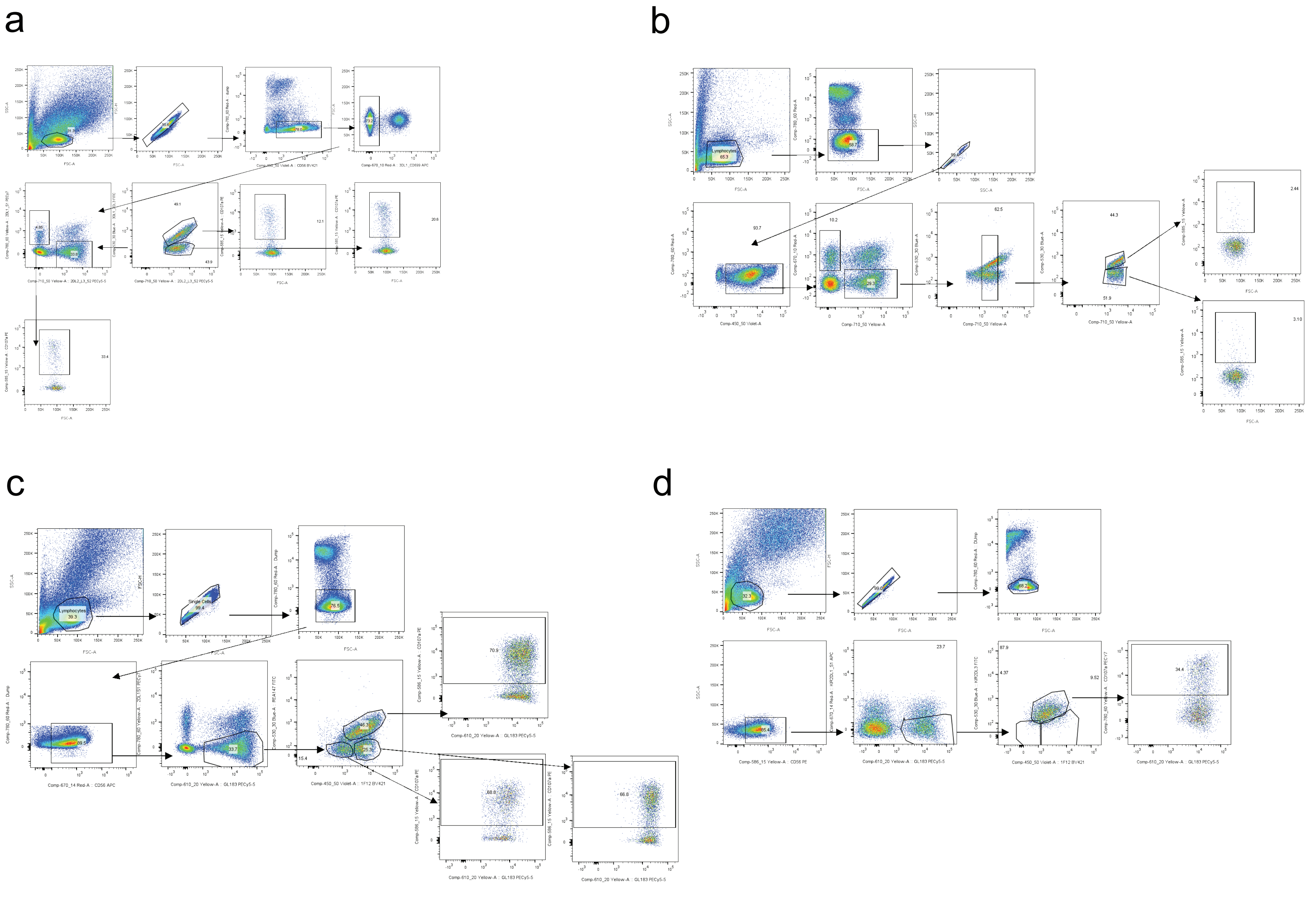

Supplementary Figure 9. Gating strategy used for NK subset analyses. (a) Gating strategy for Figure 5c and 5d. (b) Gating strategy for Figure 5f. (c) Gating strategy for Figure 6 and Supplementary Figures 8a and 8b (heterozygous). (d) Gating strategy for Supplementary Figures 8a and 8c (homozygous).

Supplementary Table.1 Data collection and refinement statistics for KIR2DL2 and 2DL3 in complex with HLAC*07:02-RL9

Data collection statistics	KIR2DL2	KIR2DL3
Temperature (K)	100	100
X-ray source	MX1<br>Australian Synchrotron	MX1<br>Australian Synchrotron
Space group	$P\ 2_1$	$P\ 6_4$
Cell Dimensions (Å)	a=68.5<br>b=82.1<br>c=104.9<br>β=90.1°	a=111.8<br>b=111.8<br>c=87.9<br>γ=120.0°
Resolution (Å)	32.32 – 3.0<br>(3.12 – 3.0)	32.57 - 2.50<br>(2.6 - 2.50)
Total no. observations	90401(8384)	131580(13083)
No. unique observations	20697 (2015)	21403 (2136)
Multiplicity	4.0 (3.8)	3.1 (3.0)
Data completeness (%)	96.7 (90.5)	98.5 (98.7)
I/σI (Average)	4.6 (1.8)	14.2 (6.8)
Rmerge[1] (%)	0.24 (0.88)	0.055 (0.23)
Refinement statistics		
Rfactor[2] (%)	0.2501	0.2263
Rfree (%)	0.2936	0.2565
Non-hydrogen atoms	9022	4517
Macromolecules	9022	4478
Protein residues	1118	553
Water	0	39
r.m.s.d. from ideality		
Bond lengths (Å)	0.004	0.003
Bond angles (°)	0.85	0.65
Ramachandran plot		
Favoured regions (%)	95	96
Allowed regions (%)	5	4
Outliers (%)	0	0
B-factors (Å2) (Average)	46.4	67.6

[1] $R_{merge} = \sum_{hkl} \sum_j |I_{hkl,j} - < I_{hkl} > | / \sum_{hkl} \sum_j I_{hkl,j}$.

[2] $R_{factor} = \sum_{hkl} ||F_o| - |F_c|| / \sum_{hkl}|F_o|$ for all data excluding the 5% that comprised the R_{free} used for cross-validation

Supplementary Table 2. KIR2DL residue contacts with HLA-C molecules

HLA residues				KIR2DL2/3 residues			
C*03:04	C*04:01	C*07:02 (2DL2)	C*07:02 (2DL3)	2DL2 (C*03:04)	2DL1 (C*04:01)	2DL2 (C*07:02)	2DL3 (C*07:02)
		Pro 20				Phe 45	
Arg 69*	Arg 69	Arg 69	Arg 69*	Glu 21*<br>Met 70	Arg 68	Glu 21*<br>Met 70	Glu 21*<br>Met 70
Gln 72*	Gln 72*	Gln 72		 Met 70<br><br>Asp 72*	Arg 68*	 Met 70<br>Gln 71<br>Asp 72	
Arg 75	Arg 75	Arg 75	Arg 75*	Phe 45<br>Asp 72	Phe 45	Phe 45	Phe 45<br>Asp 72*
Val 76	Val 76	Val 76	Val 76	 Phe 45<br>Gln 71<br>Asp 72	Met 44<br>Phe 45<br><br>Asp 72	Lys 44<br>Phe 45<br>Gln 71<br>Asp 72<br>Glu 187	 Phe 45
Arg 79	Arg 79	Arg 79*	Arg 79*	Lys 44<br>Phe 45	Met 44<br>Phe 45	Lys 44*<br>Phe 45	Lys 44*<br>Phe 45
Asn 80*	Lys 80*	Asn 80*	Asn 80*	Lys 44*<br>Ser 184	Met 44<br>Gln 71<br>Ser 184*<br>Glu 187*	Lys 44*<br>Ser 184	Lys 44*
Tyr 84	Tyr 84*	Tyr 84	Tyr 84	Asp 183	Asp 183*	Asp183	Asp 183
			Ile 142				Asp 183
Arg 145*	Arg 145*	Arg 145*	Arg 145*	Ser 133*<br>Asp 135*	Ser 133*<br>Asp 135*	Ser 133*<br>Asp 135*	Ser 133<br>Asp 135*<br>Phe 181
Lys 146*	Lys 146*	Lys 146*	Lys 146*	Tyr 105<br>Phe 181<br>Asp 183*<br>Ser 184	Tyr 105<br>Phe 181<br>Asp183*<br>Ser 184	Tyr 105<br>Phe 181<br>Asp 183*<br>Ser 184*<br>Glu 187	Tyr 105<br>Phe 181<br>Asp 183*
Ala 149*	Ala 149*	Ala 149*	Ala 149*	Tyr 105<br>Glu 106*<br>Ser 132	Tyr 105<br>Glu 106*<br>Ser 132<br>Tyr 134	Tyr 105<br>Glu 106*<br>Ser 132<br>Tyr 134<br>Phe 181	Tyr 105<br>Glu 106*<br>Ser 132<br><br>Phe 181
Ala 150	Ala 150	Ala 150	Ala 150	Leu 104<br>Tyr 105	Leu 104	Leu 104<br>Tyr 105	Leu 104<br>Tyr 105
Arg 151*	Arg 151	Arg 151		Glu 106*	Glu 106	Glu 106	
Peptide							
C*03:04	C*04:01	C*07:02 (2DL2)	C*07:02 (2DL3)	2DL2 (C*03:04)	2DL1 (C*04:01)	2DL2 (C*07:02)	2DL3 (C*07:02)
Leu 7		Val 7	Val 7	Gln 71<br>Leu 104<br>Tyr 105		Gln 71<br>Leu 104	Leu 104
Ala 8*	Lys 8	Ala 8*	Ala 8	Gln71*	Gln 71	Gln 71*	Gln 71

*Includes a H-bond or salt-bridge interaction with this residue

Supplementary Table 3. Primers for KIR2DL2 and KIR2DL3 mutagenesis.

Name	Sequence
Glu21Ala-Forward	cgcctggtgaaatcagcagagacagtcatcctg
Glu21Ala-Reverse	caggatgactgtctctgctgatttcaccaggcg
Lys44Ala-Forward	ccttctgcacagagaaggggcgtttaaggacactttgcac
Lys44Ala-Reverse	gtgcaaagtgtccttaaacgccccttctctgtgcagaagg
Phe45Ala-Forward	tctgcacagagaagggaaggctaaggacactttgcacctc
Phe45Ala-Reverse	gaggtgcaaagtgtccttagccttcccttctctgtgcaga
Met70Ala-Forward	ctccatcggtcccatggcgcaagaccttgcaggg
Met70Ala-Reverse	ccctgcaaggtcttgcgccatgggaccgatggag
Gln71Ala-Forward	catcggtcccatgatggcagaccttgcagggacc
Gln71Ala-Reverse	ggtccctgcaaggtctgccatcatgggaccgatg
Asp72Ala-Forward	gtcccatgatgcaagcccttgcagggaccta
Asp72Ala-Reverse	taggtccctgcaagggcttgcatcatgggac
Leu104Ala-Forward	ggacatcgtcatcacaggtgcatatgagaaaccttctctc
Leu104Ala-Reverse	gagagaaggtttctcatatgcacctgtgatgacgatgtcc
Tyr105Ala-Forward	gacatcgtcatcacaggtctagctgagaaaccttctctctca
Tyr105Ala-Reverse	tgagagagaaggtttctcagctagacctgtgatgacgatgtc
Glu106Ala-Forward	tcatcacaggtctatatgcgaaaccttctctctcagc
Glu106Ala-Reverse	gctgagagagaaggtttcgcatatagacctgtgatga
Ser132Ala-Forward	tcctgcagctcccgggcctcctatgacatgtac
Ser132Ala-Reverse	gtacatgtcataggaggcccgggagctgcagga
Ser133Ala-Forward	gcagctcccggagcgcctatgacatgtacc
Ser133Ala-Reverse	ggtacatgtcataggcgctccgggagctgc
Asp135Ala-Forward	tcccggagctcctatgccatgtaccatctatcc
Asp135Ala-Reverse	ggatagatggtacatggcataggagctccggga
Phe181Ala-Forward	gaacctacagatgcttcggctctgcccgtgactctcc
Phe181Ala-Reverse	ggagagtcacgggcagagccgaagcatctgtaggttc
Asp183Ala-Forward	tcggctctttccgtgcctctccatacgagtg
Asp183Ala-Reverse	cactcgtatggagaggcacggaaagagccga
