# Supplementary material for: Structural plasticity of KIR2DL2 and KIR2DL3 enables altered docking geometries atop HLA-C
Source: Nat Commun. 2021 Apr 12;12:2173. doi: 10.1038/s41467-021-22359-x (PMC8041999; doi:10.1038/s41467-021-22359-x)
Supplement: Supplementary file 3 — Reporting Summary [file 41467_2021_22359_MOESM3_ESM.pdf]

nature research

Corresponding author(s): Julian Vivian
Andrew Brooks
Jamie Rossjohn

Last updated by author(s): Feb 19, 2021

Reporting Summary

Nature Research wishes to improve the reproducibility of the work that we publish. This form provides structure for consistency and transparency in reporting. For further information on Nature Research policies, see our Editorial Policies and the Editorial Policy Checklist.

Statistics

For all statistical analyses, confirm that the following items are present in the figure legend, table legend, main text, or Methods section.

n/a	Confirmed	
☐	☒	The exact sample size (*n*) for each experimental group/condition, given as a discrete number and unit of measurement
☐	☒	A statement on whether measurements were taken from distinct samples or whether the same sample was measured repeatedly
☐	☒	The statistical test(s) used AND whether they are one- or two-sided<br>*Only common tests should be described solely by name; describe more complex techniques in the Methods section.*
☒	☐	A description of all covariates tested
☒	☐	A description of any assumptions or corrections, such as tests of normality and adjustment for multiple comparisons
☐	☒	A full description of the statistical parameters including central tendency (e.g. means) or other basic estimates (e.g. regression coefficient) AND variation (e.g. standard deviation) or associated estimates of uncertainty (e.g. confidence intervals)
☐	☒	For null hypothesis testing, the test statistic (e.g. *F*, *t*, *r*) with confidence intervals, effect sizes, degrees of freedom and *P* value noted<br>*Give P values as exact values whenever suitable.*
☒	☐	For Bayesian analysis, information on the choice of priors and Markov chain Monte Carlo settings
☒	☐	For hierarchical and complex designs, identification of the appropriate level for tests and full reporting of outcomes
☒	☐	Estimates of effect sizes (e.g. Cohen's *d*, Pearson's *r*), indicating how they were calculated

Our web collection on statistics for biologists contains articles on many of the points above.

Software and code

Policy information about availability of computer code

Data collection	All software used is publicly available. CCP4 version 7.1. Phenix version 1.10.1. FlowJo Version 10.7
Data analysis	All software used is publicly available. Phenix version 1.10.1. FlowJo Version 10.7. COOT 0.9. HLA Fusion 4.0. Molprobity 4.5.

For manuscripts utilizing custom algorithms or software that are central to the research but not yet described in published literature, software must be made available to editors and reviewers. We strongly encourage code deposition in a community repository (e.g. GitHub). See the Nature Research guidelines for submitting code & software for further information.

Data

Policy information about availability of data

All manuscripts must include a data availability statement. This statement should provide the following information, where applicable:

- Accession codes, unique identifiers, or web links for publicly available datasets
- A list of figures that have associated raw data
- A description of any restrictions on data availability

The KIR2DL2 and 2DL3-HLA-C*07:02-RL9 complexes were deposited in the Protein Databank under accession codes 6PA1 and 6PAG respectively.

Field-specific reporting

Life sciences study design

All studies must disclose on these points even when the disclosure is negative.

Sample size	No statistical methods were used to determine sample sizes. Where human donor samples were used, multiple donors were tested in separate experiments with similar trends observed which was sufficient to demonstrate functional difference.
Data exclusions	For human donor data, only donors who had identifiable KIR2DL2 and KIR2DL3 populations within a single donor were included in Figure 5. This was done to ensure variables other than stimulation conditions were not influencing the readout. No other data exclusions were done.
Replication	In most instances experiments were repeated at least twice. When data has been pooled from multiple experiments, this is indicated in the text.
Randomization	Not applicable.
Blinding	Investigators were not blinded. Not relevant for experimental setup.

Reporting for specific materials, systems and methods

We require information from authors about some types of materials, experimental systems and methods used in many studies. Here, indicate whether each material, system or method listed is relevant to your study. If you are not sure if a list item applies to your research, read the appropriate section before selecting a response.

Materials & experimental systems

n/a	Involved in the study	
☐	☒	Antibodies
☐	☒	Eukaryotic cell lines
☒	☐	Palaeontology and archaeology
☒	☐	Animals and other organisms
☐	☒	Human research participants
☒	☐	Clinical data
☒	☐	Dual use research of concern

Methods

n/a	Involved in the study	
☒	☐	ChIP-seq
☐	☒	Flow cytometry
☒	☐	MRI-based neuroimaging

Antibodies

Antibodies used

All antibodies used in the study are described in the Methods section, including Supplier, fluorochrome used, clone and dilution.
They are as follows:
CD107a PE (BD Biosciences; clone H4A3; cat 555801) Dilution 1:100
CD107a PECy5 (BD Biosciences; clone H4A3; cat 555802) Dilution 1:100
CD107a PECy7 (BD Biosciences; clone H4A3; cat 561348) Dilution 1:100
CD56 BV421 (BD Biosciences; clone NCAM16.2 cat 659452) Dilution 1:50
CD56 APC (BD Biosciences, clone B159, cat 555518) Dilution 1:50
CD56 PE (BD Biosciences, clone B159, cat 555516) Dilution 1:50
KIR3DL1 APC (BD Biosciences; clone DX9 cat 564103) Dilution 1:50
CD3 APCCy7 (BD Biosciences; clone SK7 cat 557832) Dilution 1:50
KIR2DL2/L3/S2 PECy5.5 (Beckman Coulter; clone GL183; cat A66900) Dilution 1:50-1:200
KIR2DL2/L3/S2 BUV737 (BD Biosciences; clone CH-L; cat 749479) Dilution 1:50
KIR2DL1/S1 PECy7 (Beckman Coulter; clone EB6; cat A66899) Dilution 1:50
KIR2DL1/S1 APC (Beckman Coulter; clone EB6; cat A22332); Dilution 1:50
KIR2DL3 FITC (Miltenyi Biotech; clone RAE147 cat 130-100-125) Dilution 1:50
HLA-C unlabelled (Sigma; clone DT9; cat MABF233) Dilution 1:50
Chicken anti-mouse IgG AF488 (Life Technologies, cat A21200) Dilution 1:100
Goat Anti-Human IgG PE (One Lambda, cat LS-AB2)
KIR2DL2 unlabelled clone 1F12 (Gift from Christelle Retière) Dilution 1:50
goat-anti mouse IgG BV421 antibody (Biolegend; clone poly4053; cat 405317) Dilution 1:50
W6/32 (mouse IgG) is in house hybridoma supernatant.
The Y9 blocking antibody was also a hybridoma supernatant a kind gift from Gabriella Pietra, University of Genova, Italy.

Validation

All primary staining antibodies, except w6/32 and 1F12 were sourced from commercial suppliers who have validated these antibodies for flow cytometry use as indicated on manufacturer website. The w6/32 (anti HLA -antibody, mouse IgG) was used from hybridoma supernatant. The validation for this antibody has been published (Cell, 1978 May;14(1):9-20). The validation for 1F12 antibody has been published (Immunology, 2009, 128(2): 172–184). The validation of Y9 antibody has been published (PNAS 1998; 95(3): 1172–1177).

Eukaryotic cell lines

Policy information about cell lines

Cell line source(s)	Burkitt Lymphoma cell lines (Raji, Ramos and Namalwa) were sourced from A/Prof. Paul Nesson , Peter MacCallum Cancer Centre, Melbourne, Australia. Parental 721.221 cells were sourced from John Coligan, NIH. Transfected 221 cells were generated in the laboratory of Prof. Andrew Brooks. Parental 293T cell line was sourced from Prof. Jim McCluskey, University of Melbourne, Australia. Transfected 293T cells were generated in the laboratory of Prof. Andrew Brooks.
Authentication	Cell lines have not been authenticated
Mycoplasma contamination	Cell lines were not regularly tested for mycoplasma status.
Commonly misidentified lines (See ICLAC register)	No commonly misidentified cell lines have been used in this study.

Human research participants

Policy information about studies involving human research participants

Population characteristics	Buffy packs from healthy adult volunteer donors were sourced through Australian Red Cross Blood Services. All samples are de-identified.
Recruitment	Recruitment is not relevant to this study. Healthy volunteer donor blood was sourced from Australian Red Cross Blood Services without any additional information being requested.
Ethics oversight	University of Melbourne Human Ethics Committee

Note that full information on the approval of the study protocol must also be provided in the manuscript.

Flow Cytometry

Plots

Confirm that:

☐ The axis labels state the marker and fluorochrome used (e.g. CD4-FITC).

☐ The axis scales are clearly visible. Include numbers along axes only for bottom left plot of group (a 'group' is an analysis of identical markers).

☐ All plots are contour plots with outliers or pseudocolor plots.

☒ A numerical value for number of cells or percentage (with statistics) is provided.

Methodology

Sample preparation	Human PBMC were isolated from Red Cross buffy packs and frozen in liquid nitrogen for later use. NK cells were isolated from frozen PBMCs using NK cell enrichment kit, culture overnight with 100U/ml IL-2 before the assay (as detailed in the Methods Section).
Instrument	BD Biosciences LSR II and BD Biosciences LSR Fortessa.
Software	BD Facs Diva software for collection and FlowJo v10 (TreeStar) for analysis.
Cell population abundance	NK cells comprise up to 10% of PBMC and are enriched before the assay using NK cell enrichment kit. Consistently this enriches NK cells to at least 80% purity. No flow cytometry sorting was performed for these experiments.
Gating strategy	For flow cytometric analysis of human NK cells, cells were gated on fcs/ssc to exclude debris and doublets and live/dead stain is used to eliminate dead cells from analysis. CD3 stain was used to exclude any T cells from the analysis and NK cells were identified as CD56+CD3- cells. For determining cells positive for CD107a negative population in unstimulated cells was used to delineate between a positive and negative stain. Gating strategy is shown in main Figures and as Supplementary Figure.

☒ Tick this box to confirm that a figure exemplifying the gating strategy is provided in the Supplementary Information.
